# Spatial Multiomics Analysis Reveals Only Minor Genetic and Epigenetic Changes in Human Liver Cancer Stem-Like Cells Compared With Other Tumor Parenchymal Cells

**DOI:** 10.3389/fcell.2022.810687

**Published:** 2022-02-09

**Authors:** Dan Liu, Hong Li, Hui Dong, Mincheng Qu, Liguang Yang, Lina Chen, Yixue Li, Hongyang Wang, Yufei He

**Affiliations:** ^1^ Model Animal Research Center, Medical School, Nanjing University, Nanjing, China; ^2^ Molecular Pathology Laboratory, National Center for Liver Cancer, Eastern Hepatobiliary Surgery Hospital, Shanghai, China; ^3^ CAS Key Laboratory of Computational Biology, Shanghai Institute of Nutrition and Health, University of Chinese Academy of Sciences, Chinese Academy of Sciences, Shanghai, China; ^4^ Department of Pathology, Eastern Hepatobiliary Surgery Hospital, Shanghai, China; ^5^ National Center for Liver Cancer and International Cooperation Laboratory on Signal Transduction, Eastern Hepatobiliary Surgery Institute/Hospital, Shanghai, China

**Keywords:** DNA methylation, cytosine modification, CIL-seq, exome sequencing, spatial transcriptome

## Abstract

Cancer stem cells (CSCs) usually account for a very small tumor cell population but play pivotal roles in human cancer development and recurrence. A fundamental question in cancer biology is what genetic and epigenetic changes occur in CSCs. Here we show that the *in-situ* global levels of DNA cytosine modifications, including 5-methylcytosine (5mC), 5-hydroxymethylcytosine (5hmC) and 5-formylcytosine (5fC), are similar between liver cancer stem-like (LCSL) cells and paratumor liver cells of liver cancer patients. We then developed a robust method combining immunohistochemistry, laser capture microdissection and genome sequencing with ultra-low-input cells (CIL-seq) to study the detailed genetic and DNA methylation changes in human LCSL cells. We first used clinical samples of mixed hepatocellular carcinoma-cholangiocarcinoma (HCC-CCA) with stem cell features to investigate human LCSL cells. The CIL-seq analysis of HCC-CCA and HCC patients showed that LCSL cells had strong spatial genetic and epigenetic heterogeneity. More interestingly, although the LCSL cells had some potential key changes in their genome, they had substantially fewer somatic single nucleotide variants (SNVs), copy number alterations (CNAs) and differentially methylated regions than other tumor parenchymal cells. The cluster analysis of SNVs, CNAs, DNA methylation patterns and spatial transcriptomes all clearly showed that the LCSL cells were clustered with the paratumor liver cells. Thus, spatial multiomics analysis showed that LCSL cells had only minor genetic and epigenetic changes compared with other tumor parenchymal cells. Targeting key changes in CSCs, not just changes in bulk tumor cells, should be more effective for human cancer therapy.

## Introduction

Human cancer development is a complex evolutionary process (Greaves and Maley, 2012; Black and McGranahan, 2021). To date, it is universally acknowledged that cancer cells possess many significant genetic and epigenetic changes caused by multiple factors compared with normal cells. Some of these changes are essential for tumor evolution (Black and McGranahan, 2021). Many cancers are thought to originate from cancer stem cells (CSCs), which pose a high risk of therapy resistance and cancer relapse (Greaves and Maley, 2012; Black and McGranahan, 2021). Understanding the genetic and epigenetic changes in human CSCs should shed light on a better understanding of the developmental and evolutionary trajectory of a tumor and the design of better cancer therapeutic approaches. However, it is always difficult to study human CSCs, as they generally account for a very small proportion of tumor specimens from clinical patients. Using cell culture and animal models, including the xenotransplantation approach, to investigate the properties of CSCs has inherent technical and conceptual limitations (Batlle and Clevers, 2017). Consequently, the genetic and epigenetic changes that occur in human CSCs are still poorly understood.

Liver cancer is one of the most common cancer types worldwide. In the current study, we developed a robust spatial multiomics method for genome sequencing with ultra-low-input cells, to uncover the genetic and DNA methylation changes related to human CSCs with different spatial locations. We first selected specimens with a stem cell phenotype from mixed hepatocellular cholangiocarcinoma (HCC-CCA) patients to investigate liver cancer stem-like (LCSL) cells. We also investigated LCSL cells in other liver cancer samples using spatial multiomics analysis.

## Materials and Methods

### Patients and Collection of Clinical Specimens

#### Formalin-Fixed, Paraffin-Embedded (FFPE) Specimens

For mixed HCC-CCA cases collection, pathological sections of 12,603 primary liver cancer (PLC) patients were investigated who received surgical resection from 2017 to 2019 at the Eastern Hepatobiliary Surgery Hospital (EHBH) in Shanghai, China, and finally total 278 HCC-CCA cases were found out and further investigated for LCSL cells. In accordance with the number of HCC-CCA cases, 265 HCC patients who underwent surgical resection from 2009 to 2020 at EHBH were randomly selected and investigated for LCSL cells (Supplementary Table S1).

#### Fresh Frozen Specimens

Fresh PLC tissues and matched paratumor liver tissues were obtained from 58 patients (35 cases in cohort 1 and 23 cases in cohort 2) who underwent surgical resection at EHBH (Supplementary Table S1). Each tissue specimen was embedded in optimal cutting temperature (OCT) compound medium, immediately frozen in an isopentane slurry made with liquid nitrogen, and finally stored at −80°C until further processing. Each tissue was embedded within 30 min for frozen sectioning after surgical removal. All histological specimens were evaluated by at least two experienced pathologists.

The archives of all patients were collected by the EHBH archive system. Informed consent was obtained from the patients, and all procedures were approved by the ethical committee of EHBH.

### Immunohistochemistry (IHC)

For IHC staining of frozen sections, briefly, OCT-embedded frozen tissues were cut into 8 µm thick sections, and endogenous peroxidases were inactivated by incubation in 0.3% H_2_O_2_. Nonspecific signals were blocked using 5% bovine serum albumin. The primary antibodies used were as follows: anti-EpCAM (1:500, ab7504; Abcam), anti-OV6 (1:200, MAB2020; R&D Systems) and anti-GPC3 (1:100, ab207080; Abcam). HRP-conjugated antibodies were used as the secondary antibodies. Diaminobenzidine colorimetric reagent solution was used for staining followed by hematoxylin counterstaining. The slides were finally scanned, and representative images were illustrated.

For IHC staining of FFPE sections, the tissues were fixed overnight in 4% PFA, embedded in paraffin and cut into 4 μm serial consecutive sections. After deparaffinization, antigen retrieval was performed with sodium citrate buffer (10 mM sodium citrate, 0.05% Tween-20, pH 6) (for H&E, omit this step, subsequently by hematoxylin and eosin standard protocols). The following steps were the same as those in the aforementioned frozen section. The primary antibodies used here were as follows: anti-SALL4 (1:100, sc-101147; Santa Cruz Biotechnology), anti-CK19 (1:100, ab9221; Abcam), and anti-Ki-67 (1:1000, ab15580; Abcam).

For 5mC, 5hmC or 5fC immunostaining, DNA was denatured with 2 N HCl for 15 min at room temperature after antigen retrieval treatment, followed by neutralization with 100 mM Tris-HCl (pH 8.0) for 10 min at room temperature. The primary antibodies used were as follows: anti-5mC (1:5000, Cat#: 28692; Cell Signaling Technology), anti-5hmC (1:10000, Cat#: 39769; Active Motif); and anti-5fC (1:1000, Cat#: 61227; Active Motif). For EpCAM and 5hmC double-staining, primary anti-5hmC antibody (rabbit) was first incubated for 16 h at 4°C, followed by alkaline phosphatase–based streptavidin/biotinylated link system to visualize dark purple cell nuclei. Anti-EpCAM antibody (mouse) was then incubated for 2 h at room temperature, followed by detection using a horseradish peroxidase/AEC system to visualize the red cell membrane. Specifically, the slides were sealed with glycerol mounting medium and without hematoxylin counterstaining. We could not obtain good results for EpCAM and 5fC double staining, possibly because of the low 5fC content in cells and thus usually weak staining for 5fC.

### CIL-Seq

#### IHC and Laser Capture Microdissection (LCM)

OCT-embedded frozen tissues were cut into 10 µm thick sections and mounted on PEN Membrane Glass Slides (Leica). After IHC of EpCAM (*see* above), tissues were dehydrated through rising ethanol concentrations (50, 75, 85, 95, 100 and 100% ethanol, 60 s each) in 50 ml sterile centrifuge tubes. When membrane slides dried completely, the LCM procedure was initiated by a Leica LMD 7000 laser microdissection system according to the manufacturer’s protocol. Two pathologists confirmed the different tumor or paratumor components independently. All captured samples were collected in 0.2 ml PCR tubes. Finally, the membrane slides were cleared with xylene and sealed for long-term preservation. Certified RNase/DNase-free materials were used whenever available.

#### Genetic Libraries

Ultralow DNA from LCM samples was extracted by a Quick-DNA Microprep Plus Kit (Zymo Research) before single-cell multiple displacement amplification (MDA) using a REPLI-g Single Cell Kit (Qiagen) for trace DNA amplification. After obtaining high yields of high-quality whole-genome amplified DNA, whole-exome capture and library construction were carried out by an Agilent SureSelect Human All Exon V6 kit (Agilent) according to the manufacturer’s instructions. Whole-genome libraries were constructed by the QIAseq FX Single Cell DNA Library kit (Qiagen).

#### Whole-Genome Bisulfite Sequencing (WGBS) Libraries

Tissue samples from LCM were digested for up to 4 h with 1 mg/ml proteinase K according to the EZ DNA Methylation-Direct Kit (Zymo Research). After thorough digestion, LCM cells were immediately sent to the Pico Methyl-Seq Library Prep Kit (Zymo Research) to accommodate ultralow DNA input according to the manufacturer’s instructions with minor adjustment: bisulfite conversion time was extended to 90 min to ensure complete conversion; for the sample M1BULK, the library only needed six cycles in Section 4, while other libraries needed 11 cycles.

#### Sequencing

All libraries were sequenced with the Illumina Xten platform to generate 150 bp paired-end reads.

### Whole-Genome Sequencing (WGS) and Whole-Exome Sequencing (WES) Data Analysis

#### Data Quality and Filtering

Raw reads were filtered with Trimmomatic to remove adaptor sequences and low-quality bases (Bolger et al., 2014). The remaining reads were aligned to the human genome (hg38) by the BWA algorithm (Li and Durbin, 2009). After mapping, the duplicate reads were removed. Germline variants were called using GATK (Auwera et al., 2013).

#### Somatic Mutations

Taking bulk immune infiltrating cells as a control, somatic mutations were identified from BAM files using muTect and varScan (Koboldt et al., 2012; Cibulskis et al., 2013). High-quality somatic single nucleotide variants (SNVs) were obtained by applying the following filters: *1*) Removal of potential germline variants that are recorded in dbSNP or variants with allele frequency larger than 1% in population (Sherry et al., 2001; Genomes Project Consortium et al., 2015); *2*) Removal of variants located within 10 bp distance, which are more possible to be errors introduced during library preparation; *3*) Removal of mutations that are identified by only one algorithm; *4*) Depth of the mutation sites should be larger than 10 in sample j and control sample; *5*) There are at least two reads supporting the alternative allele and the allele frequency is larger than 5% in sample j , and no reads have the alternative allele in control sample; *6*) Since there are more samples in P2 than in P7, only mutations that occur in more than one of P2 samples are analyzed to improve quality. This filter is not suitable for P7.

Functional consequences of somatic SNVs were annotated by ANNOVAR (Wang et al., 2010). Somatic SNVs of all samples were converted to a 0–1 matrix (M) , in which 1 indicates having a mutation. To reduce the effects of allele dropout, when site i had low coverage (≤5×) in the sample, $M_{ij}$ was adjusted based only on other samples of the same pathology group. $M_{ij}=0.9$ if more samples carry the mutation or $M_{ij}=0.1$ if more samples are wild type. Euclidean distances between paired samples were calculated from M and hierarchical clustering was performed to cluster samples.

#### Copy Number Alterations

Copy number alterations (CNAs) were identified from WES data by CNVkit (Talevich et al., 2016). Samples covering <50% target regions were removed from the CNA analysis. Deletions were ignored to avoid the effects of uncovered regions. Segments and genes with log_2_ (CN/2) ≥1 were regarded as amplifications. To decrease false positives of amplified genes, we only analyzed genes that were amplified in ≥5% TCGA liver cancer patients (https://www.cancer.gov/tcga) and genes with three more amplifications in tumor samples than in paratumor samples. Euclidean distances were calculated from amplified genes, and hierarchical clustering was used to plot the clustering tree.

### WGBS Data Analysis

#### Data Quality and Filtering

Raw reads were filtered with Trimmomatic to remove adaptor sequences and low-quality bases (Bolger et al., 2014). The remaining reads were aligned to the human genome (hg38) by Bismark (Krueger and Andrews, 2011). Potential PCR duplicates were removed. The bisulfite conversion rate was estimated as the ratio between the number of methylated non-CpGs and total non-CpG sites.

#### Data Processing

The R package methylKit was used to generate and analyze the methylation matrix (Akalin et al., 2012). Bases whose coverage was larger than 500× or lower than 3× were discarded. The genome was split into 300-bp windows with 300-bp step size for fresh-frozen samples. For FFPE samples, the genome was split into 1 Mb windows with a 1 Mb step size. Windows that were covered by less than three bases were discarded. Similarity between paired samples was measured by Pearson correlation coefficients. Hierarchical clustering was used to explore the clusters of samples. For differential methylation analysis between two groups, the chi-square test was used to compare windows covered by at least two samples in each group. Differentially methylated regions (DMRs) were windows that satisfied *Q*-value < 0.05 and absolute difference >0.25. DMRs were annotated with genomic contexts and 15 chromatin states of embryonic stem cell line H9 (downloaded from Roadmap Epigenomics Project) (Roadmap Epigenomics Consortium et al., 2015). Annotation enrichment analysis was performed by Fisher’s exact test. Annotations with *Q*-values <0.01 were regarded as significantly enriched.

### Gene Ontology (GO) Enrichment Analysis

The Metascape web-based tool (Zhou et al., 2019) was used for GO analysis of the genes of interest. The Metascape analysis was performed using the default settings. A value of *p* < 0.05 was considered statistically significant.

### DNA Modification Analysis

For statistical analysis of 5mC, 5hmC and 5fC staining, we set a standard assessment rule: within one patient, the strongest stained areas were marked with three scores. Specifically, strong positive, score = 3; medium positive, score = 2; weak positive, score = 1; no signal, score = 0. Five randomly selected equal fields (400×) of IHC images of each cell type were analyzed.

### Spatial transcriptome Analysis

#### ST Experiments

Spatial transcriptome (ST) experiments were performed according to the user guide of Visium Spatial Gene Expression Reagent Kits (10× Genomics). Briefly, HCC tissues were gently washed with cold PBS and filled with OCT in a proper mold and then snap frozen in chilled isopentane. Cryosections were mounted onto a spatially barcoded array of ST with 10-μm thickness, and serial adjacent cyosections were mounted onto regular glass slides for IHC staining of EpCAM or other markers with 8-μm thickness. For processing, the tissue was fixed for 30 min with prechilled methanol at −20°C, followed by H&E staining. Slides were finally taken on a Leica SCN400 F whole-slide scanner at 40× resolution. After capturing ideal tissue morphology information and ensuring that RNA was not degraded (RIN ≥7), tissue permeabilization and reverse transcription were immediately conducted by a Visium Spatial Tissue Optimization Kit (10× Genomics). Finally, the ST library was prepared with second strand synthesis and denaturation and sequenced by NovaSeq 6000 (Illumina). Each of the spots printed onto the array is 50 μm in diameter and 100 μm from center to center, covering an area of 6.5 × 6.5 mm^2^ (ref. (Ståhl et al., 2016)).

#### Data Processing and Cluster Analysis

Raw sequenced ST reads were processed using Space Ranger analysis software (version 1.0.0, 10× Genomics) mapped to the GRCH38 genome assembly following the standardized analysis rules. Unique molecular identifier counts in each spot were normalized and scaled by the median number transcript count across all spots. Cluster analysis was based on the K-Means algorithm in Space Ranger software.

### Statistical Analyses

Statistical analysis and data visualization were carried out using the R/Biocoductor software packages (http://www.bioconductor.org) or GraphPad Prism 8 (GraphPad Software, La Jolla, CA, USA). All *p*-values less than 0.05 were considered statistically significant.

## Results

### Characteristics of LCSL Cells in Liver Cancer Patients

Mixed hepatocellular cholangiocarcinoma (HCC-CCA) is a rare type (less than 5%) of primary liver cancer (PLC) with mixed differentiation (Supplementary Figure S1A), and its diagnosis depends on postoperative histopathology (Brunt et al., 2018). Some cancer cells in mixed HCC-CCAs have stem cell characteristics (Brunt et al., 2018; Munoz-Garrido and Rodrigues, 2019). The proportion of CSCs or LCSL cells in mixed HCC-CCAs is usually much higher than that in other types of tumors. Therefore, this type of PLC can provide us with a perfect tumor object directly from clinical patients to help investigate human CSCs or LCSL cells and compare the similarities and differences between LCSL cells and HCC or CCA cells within the same liver cancer sample. We collected fresh liver cancer specimens that were resected and diagnosed with PLC before surgery. According to the clinicopathological diagnosis, one mixed HCC-CCA case (patient P2, Supplementary Table S1) among a total of 35 cases (Supplementary Table S1) collected was identified. This mixed HCC-CCA specimen is the stem-cell feature type, which is the exact type of specimen that we aimed to research. Pathological histocytology (Supplementary Figure S1B) showed three distinct types of parenchymal cells coexisting in the tumor tissues: HCC cells, CCA cells, and LCSL cells. LCSL cells express liver stem cell markers, including epithelial cell adhesion molecule (EpCAM), cytokeratin 19 (CK19), OV6 and Sal-like protein 4 (SALL4), and account for approximately 10% of the overall tumor tissues (Supplementary Figure S2). Some LCSL cells grew in clonal clusters, and the cell morphology within each clone was not distinctly different under the microscope (Supplementary Figure S1B). Thereafter, we selected EpCAM, which was used to label liver stem cells (Huch et al., 2015; Aizarani et al., 2019) or liver CSCs (Yamashita et al., 2009; Yamashita et al., 2013; Matsumoto et al., 2017), as a marker molecule for LCSL cells or other liver stem-like cells in this study.

**FIGURE 1:**
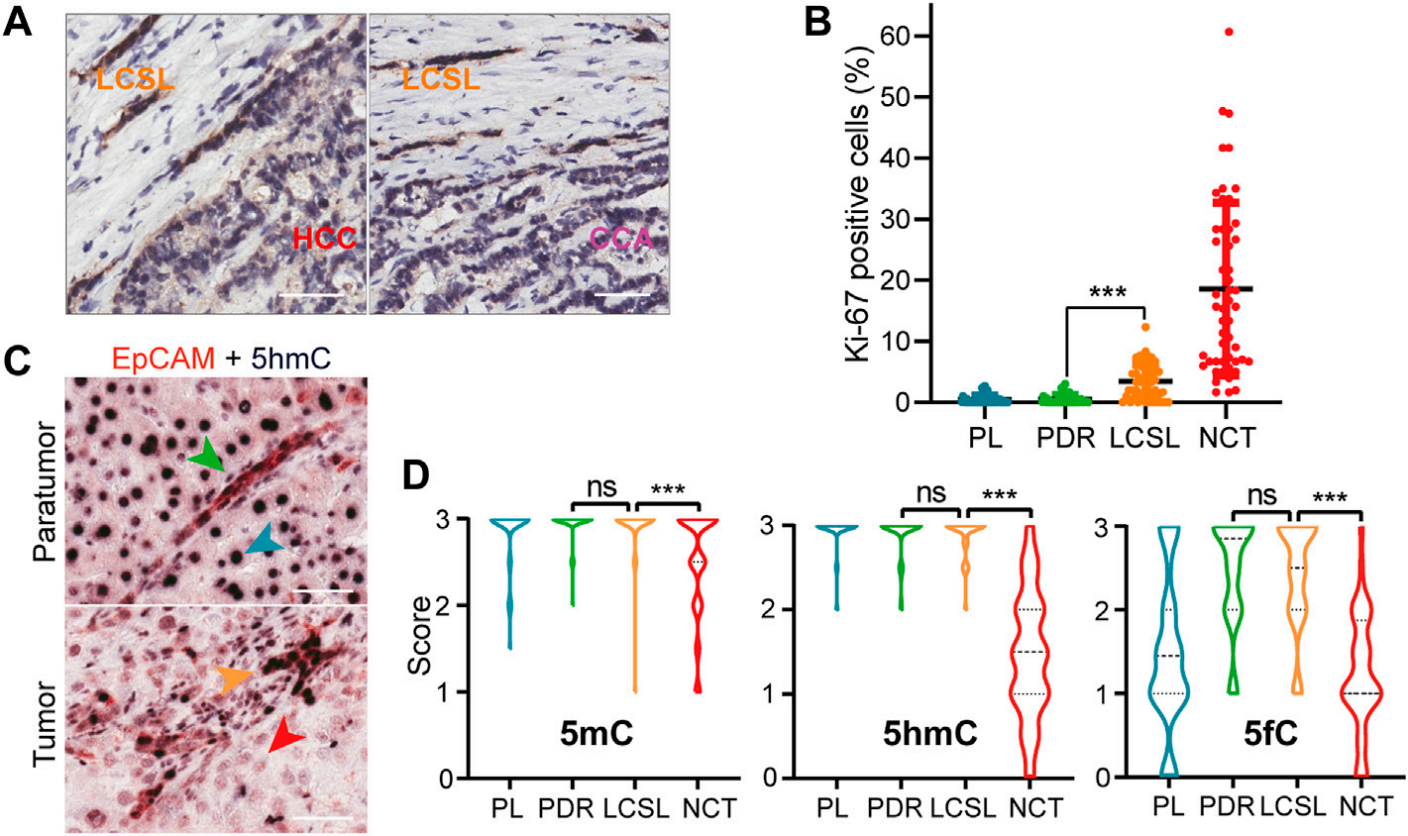
Characterization of LCSL cells in liver cancer patients. **(A)** Representative transition regions at the junction of LCSL cells and HCC cells or CCA cells (EpCAM immunostaining) in P2 tumor tissues. Scale bar, 50 μm. **(B)** Dot plot shows the percentages of Ki-67-positive cells in the four cell types of 50 PLC patients with obvious LCSL cells (Supplementary Table S1). Each dot represents the mean value of five randomly selected equal fields of IHC images from one patient. NCT, non-CSC tumor cells. Data are presented as the mean ± SD. ***, *p* < 0.001 by Wilcoxon matched-pairs signed rank test. **(C)** Representative double immunostaining of EpCAM (red) and 5hmC (dark purple) in one HCC sample with LCSL cells. The green, blue, yellow and red arrowheads indicate PDR, PL, LCSL and HCC cells, respectively. Scale bar, 50 μm. **(D)** Statistical analysis of 5mC, 5hmC and 5fC staining of liver cancer samples. NCT, non-CSC tumor cells. For 5mC and 5hmC, *n* = 65; for 5fC, *n* = 34. Data are presented as violin plots with horizontal bars indicating the mean ± SD. ***, *p* < 0.001 by Wilcoxon matched-pairs signed-rank test; ns, not significant.

By integrating EpCAM staining and tissue/cell morphology, the liver parenchymal cells in the paratumor tissue of P2 could be further subdivided into two types: the most common type was EpCAM-negative cells, which are ordinary paratumor liver (PL) cells, and the other type was EpCAM-positive cells, which correspond to paratumor ductular reaction (PDR) cells (Supplementary Figure S1B). In PDR cells, each clone is generally believed to contain a portion of liver stem cells (Sato et al., 2019). Therefore, EpCAM immunostaining combined with tissue/cell morphology features could clearly distinguish the two types of liver parenchymal cells in paratumor tissues and the three types of parenchymal cells in tumor tissues. At the junction between LCSL cells and HCC cells or CCA cells, some transitional areas could be clearly observed (Figure 1A), supporting that HCC and CCA cells may be derived from LCSL cells.

LCSL cells and PDR cells had similar characteristic markers, such as EpCAM, CK19, OV6, and SALL4 (Supplementary Figure S1B). Therefore, LCSL cells closely resembled PDR cells in both histomorphology and key markers of liver stem cells. We investigated the pathological sections of 12,603 PLC patients who received surgical resection from 2017 to 2019 at our hospital. Among them, 278 cases (2.2%) were pathologically diagnosed as HCC-CCA and 58 cases of them (Supplementary Table S1) could be observed such LCSL cells as in patient P2. We also investigated 265 HCC patients and found that 35 cases of HCC (Supplementary Table S1) could be observed such LCSL cells, whereas the number of LCSL clones was much less than that in HCC-CCA. Notably, however, LCSL cells had significantly more Ki-67-positive staining than PDR cells (Figure 1B and Supplementary Figure S1B), indicating that LCSL cells have greater proliferative activity.

### Only Minor Global DNA Cytosine Modification Changes Occur in Human LCSL Cells Compared With Other Tumor Parenchymal Cells

An important epigenetic change during cancer development is the global demethylation of DNA. We and others have found that 5-methylcytosine (5mC) can be oxidized to the demethylation intermediates 5-hydroxymethylcytosine (5hmC), 5-formylcytosine (5fC) and 5-carboxylcytosine (5caC) in mammalian cells (He et al., 2011; Xu and Bochtler, 2020). Recently, we found that in the early stage of HCC, the global content of 5hmC and 5fC was decreased (Liu et al., 2019), but it is not clear whether there is such a change in LCSL cells. We immunostained the FFPE section samples of P2 tissues for 5mC, 5hmC and 5fC. Surprisingly, the results showed that most LCSL cells were different from other tumor parenchymal cells which had obviously decreased 5hmC and 5fC staining (Supplementary Figure S3). Examination of 65 specimens with obvious LCSL cells (Supplementary Table S1), including 30 mixed HCC-CCAs and 35 HCC patients, showed similar results: there was little difference in 5mC, 5hmC or 5fC staining between LCSL cells and PDR cells (Figures 1C,D), suggesting that LCSL cells do not undergo extensive DNA demethylation. These results suggest that only minor global DNA cytosine modification changes occur in human LCSL cells.

### Development of CIL-Seq Method

**FIGURE 2:**
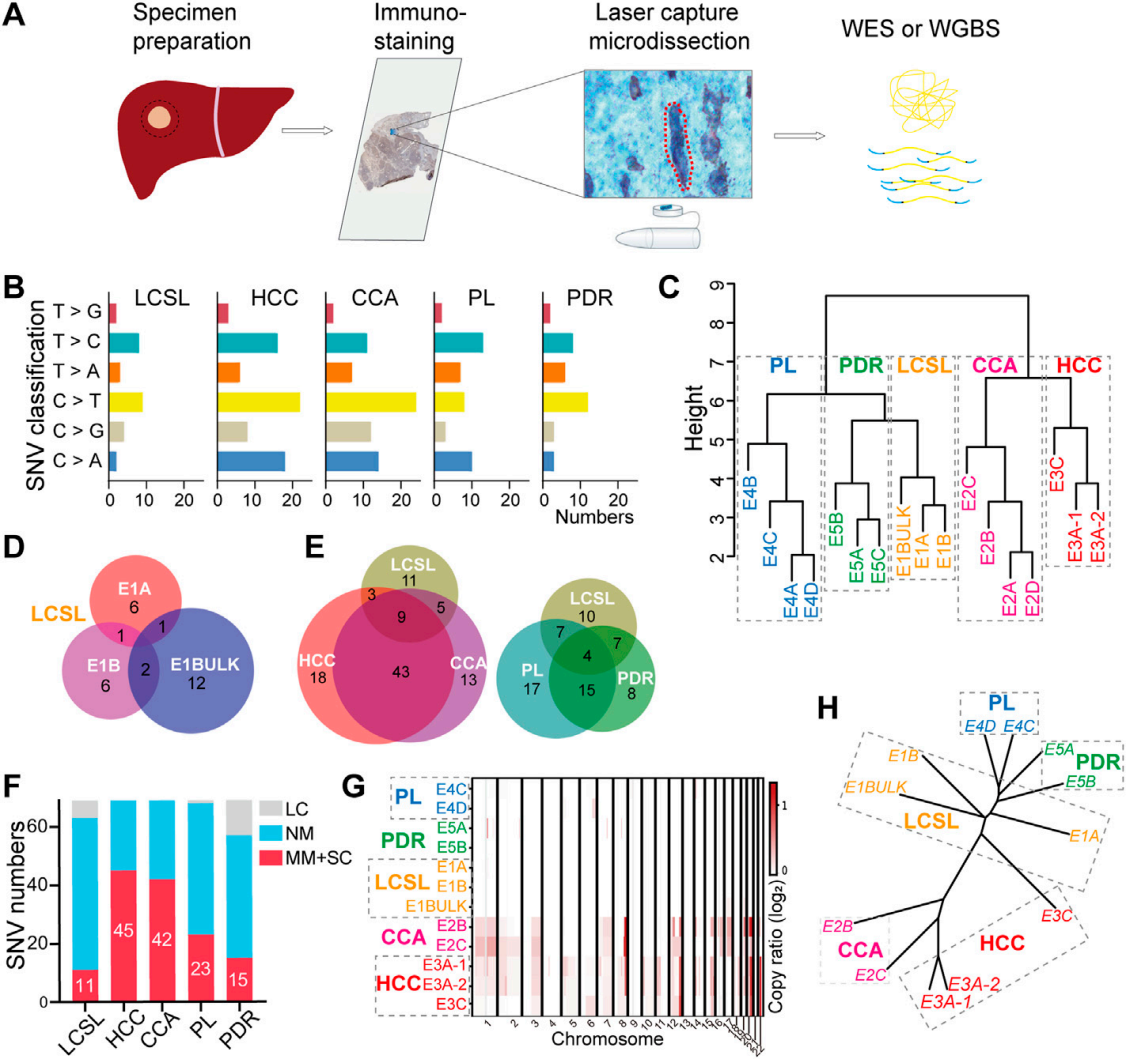
Substantially fewer genetic changes in LCSL cells than in HCC and CCA cells. **(A)** Experimental design and the CIL-seq method developed in this study (*see* also Materials and Methods). **(B)** Mutation characteristics of different cell types from P2. **(C)** SNV cluster analysis of the 16 sequencing samples from P2. **(D)** Venn diagrams show the SNVs shared among different samples of LCSL cells. *p*-value for the overlapping was calculated by hypergeometric distribution test. *p* > 0.05 in three pairwise comparisons. **(E)** Venn diagrams show the SNV overlap between LCSL cells and other types of cells. *p* > 0.05 in all pairwise comparisons, but *p* < 0.001 in HCC vs. CCA. **(F)** The number of missense mutations (MM) and stop codon changes (SC) identified in different cell types. LC, low coverage; NM, no mutation. **(G)** Heatmap shows the distribution pattern of amplified CNA regions (log_2_R > 0) on chromosomes in different samples. **(H)** Clustering evolution analysis of gene amplification in CNAs in the indicated samples. Samples with low WES coverage are not shown in **(G**,**H)**.

To explore the detailed genetic and epigenetic differences between LCSL cells and other EpCAM-stained cells in both tumor and paratumor tissues, we developed a spatial multiomics method combining immunohistochemistry (IHC), laser capture microdissection (LCM) and genome sequencing with ultra-low-input cells (CIL-seq) (Figure 2A, Materials and Methods). First, to better obtain LCSL cells for further study, we carried out EpCAM IHC staining on frozen sections. Second, we used LCM to capture needed cells more accurately and visually in multiple spatial regions, which is helpful for the investigation of tumor biology and heterogeneity. Then, to test whether the IHC process damages the integrity of genomic DNA, we selected approximately 40 pure HCC or PL cells from specimen P1 (Supplementary Figure S4A and Supplementary Table S1) for whole genome sequencing (WGS) library analysis based on single-cell technology. Simultaneous sequencing of several same-type cells could reduce the inherent single-base false positives caused by whole-genome amplification of single cells as much as possible. Last, the WGS results showed satisfactory coverage; at a 15× mean sequencing depth, WGS covered approximately 78–80% of the genome at ≥1× coverage (Supplementary Table S2), confirming the integrity of genomic DNA.

### Whole-Exome Sequencing (WES) Analysis of LCSL Cells Reveals Only Minor Genetic Changes

We performed a WES analysis of specimen P2 based on CIL-seq. According to the aforementioned EpCAM staining and tissue/cell morphology, we isolated five types of cell samples (Supplementary Figures S4A–C) from frozen sections, including three components in the tumor: LCSL cells (three samples), CCA cells (four samples) and HCC cells (two samples), and two components in the paratumor: PDR cells (three samples) and PL cells (four samples). In addition, we used bulk immune infiltrating cells (∼500 cells) from the paratumor tissue (Supplementary Figure S4B) in this patient as the normal genomic reference. Taken together, WES libraries were prepared from a total of 17 samples and sequenced. One of the 17 samples, E3A, was sequenced repeatedly, and the results showed good sequencing concordance (Supplementary Figure S4D). At least two samples of each type had good coverage (average target depth >62×) (Supplementary Table S2).

We utilized two algorithms to identify somatic single nucleotide variants (SNVs) and removed false positives by commonly used filters (Materials and Methods). Consequently, we detected 125 SNVs in all samples, and Sanger sequencing further confirmed the accuracy of SNV detection (Supplementary Table S3). They were predominantly C to T transitions (Figure 2B), similar to those previously reported (Marquardt et al., 2015; Blokzijl et al., 2016; Zhu et al., 2019). Through cluster analysis of the 125 SNVs, it was clearly seen that different samples from the same cell types could be clustered together. Intriguingly, the LCSL samples were clustered with the paratumor samples instead of the HCC or CCA samples (Figure 2C).

Most SNVs were detected in HCC or CCA samples (73/125 and 70/125, respectively, Supplementary Table S3), and the number of SNVs was consistent with that of previous findings in HCC or mixed HCC-CCA samples (Schulze et al., 2015; Lin et al., 2017; Wang et al., 2018; Xue et al., 2019). Notably, only minor SNVs (28 in total) were detected in LCSL cells. There were considerably more independent SNVs among LCSL samples (Figure 2D, Supplementary Figure S5A and Supplementary Table S3), suggesting that LCSL cells harbor strong genetic heterogeneity. HCC and CCA cells shared 52 SNVs (Figure 2E and Supplementary Table S3), indicating a monoclonal origin, which is in agreement with results from previous reports on the monoclonal origin of mixed HCC-CCA (Wang et al., 2018; Xue et al., 2019). More than half of the mutations in LCSL cells (17/28) coincided with those in HCC and CCA cells (Figure 2E and Supplementary Table S3), supporting the same origin of LCSL cells and HCC or CCA cells. Previous reports showed extensive SNVs in normal or cirrhotic liver cells (Brunner et al., 2019; Zhu et al., 2019). Consistent with these findings, we found many SNVs (43 in total, Supplementary Table S3) in PL cells.

Of the 69 genes with missense mutations or stop codon changes, only a small portion of these SNVs were detected in LCSL cells (Figure 2F and Supplementary Table S3). We found that three genes (*DHX9*, *ERBB4*, *FAM46D*) were among the 299 reported driver genes (Bailey et al., 2018), and one (*ZFHX4*) was among the 161 driver genes of HCC (Schulze et al., 2015). These driver gene mutations were detected only in HCC or CCA cells (Supplementary Table S3). Taken together, these results suggest that crucial gene mutations that may promote tumor growth have occurred in HCC and CCA cells; however, substantially fewer mutations occurred in LCSL cells.

### Only Few Key Genetic Changes in LCSL Cells Compared With Other Tumor Parenchymal Cells

We next analyzed the copy number alteration (CNA) using the WES data. Our analysis focused only on the amplified loci because of the low coverage of some samples. We found many CNA amplification regions in the tumor, but most of them were centralized in HCC and CCA cells, including the amplification of chromosome 8q, which is often detected in liver cancer (Zhai et al., 2017; Cancer Genome Atlas Resea, 2017; Xue et al., 2019), while only very few CNA regions were detected in LCSL cells (Figure 2G and Supplementary Table S4). We also analyzed the amplified genes. Overall, consistent with the CNA amplification regions, at the gene level, LCSL cells also had apparently fewer amplification genes than HCC and CCA cells (Supplementary Figure S5B and Supplementary Table S4). A phylogenetic tree constructed using the amplified genes clearly showed that similar to SNVs, LCSL cells were more similar to paratumor cells than to HCC and CCA cells (Figure 2H). Interestingly, some of these amplified genes are oncogenes, such as *MYC*, which was also amplified in one sample of LCSL cells (Supplementary Figure S6 and Supplementary Table S4). Activation of *MYC* is thought to be related to the formation of CSCs in the liver (Chow et al., 2012; Marquardt et al., 2015). Taken together, WES data showed that LCSL cells have only a few SNVs and CNAs compared to those in HCC and CCA cells, but LCSL cells might possess few crucial changes, such as the amplification of the oncogene *MYC*, which may be important for the proliferation of LCSL cells during cancer development and evolution.

### Whole-Genome Bisulfite Sequencing (WGBS) Analysis of LCSL Cells Reveals Only Minor Epigenetic Changes

Epigenetic aberrations play an important role in cancer development (Flavahan et al., 2017; Vicente-Dueñas et al., 2018; Rozenblatt-Rosen et al., 2020; Black and McGranahan, 2021), and our WES data showed only a few striking genetic changes in LCSL cells. Thus, we are curious about what epigenetic changes are hidden in LCSL cells. Considering the actual situation that the input DNA amount of a pure cluster of LCSL cells captured by LCM was very low (only ∼100 pg), we mainly examined the DNA methylation landscape of these samples. DNA methylation is the most essential DNA modification and is closely associated with other epigenetic contents (REC et al., 2015). We performed WGBS analysis of captured different cell clones (Supplementary Figure S4) from specimen P2 using CIL-seq. The bisulfite conversion efficiency of most samples was greater than 98%, and CpG coverage in most samples (Supplementary Table S2) was higher than that reported for single-cell WGBS sequencing (Smallwood et al., 2014; Gravina et al., 2016; Linker et al., 2019).

**FIGURE 3:**
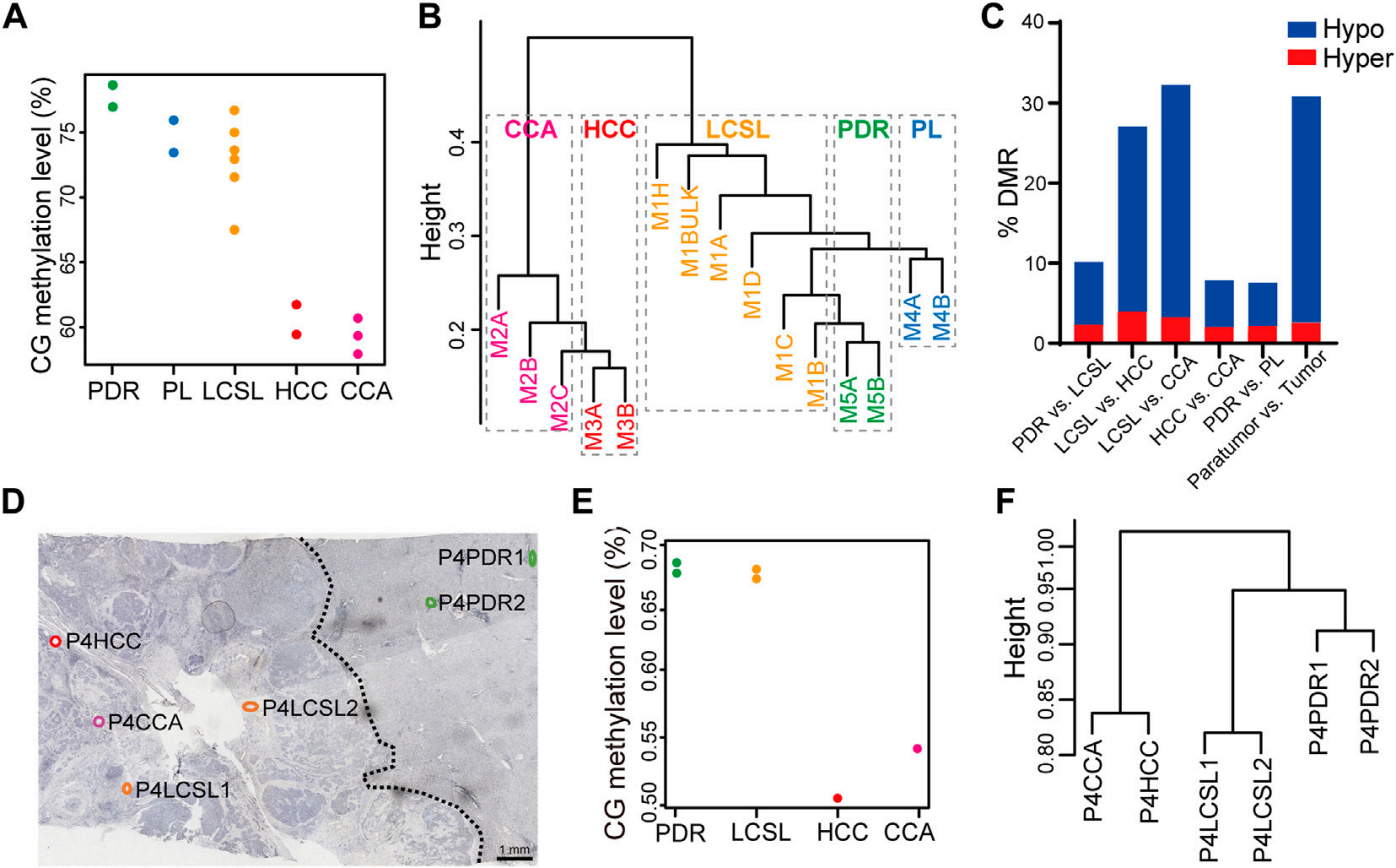
Substantially fewer DNA methylation changes in LCSL cells than in HCC and CCA cells. **(A**–**C)** WGBS analysis of different cell samples from P2. **(A)** The global DNA methylation level of five different types of cells. Each dot represents one sample. **(B)** Cluster analysis of the DNA methylation pattern of all WGBS samples. **(C)** DMR frequency between different cell types. **(D**–**F)** WGBS analysis of the FFPE HCC-CCA specimen P4. Different types of cells were captured from FFPE sections of specimen P4 and WGBS was performed by CIL-seq. **(D)** Locations and names of the samples. The section was immunostained with anti-EpCAM before LCM. The dotted lines in the figure indicate the boundaries between the tumor and the paratumor, which were determined by IHC staining and tissue/cell morphology under microscope. **(E)** The global DNA methylation levels of six different types of cells. Each dot represents one sample. **(F)** Cluster analysis of the DNA methylation patterns of all WGBS samples.

In general, HCC and CCA cells have undergone broad DNA demethylation compared with paratumor cells (Figure 3A). However, the global methylation level of LCSL cells changed much less (Figure 3A), indicating a closer relationship to paratumor cells, consistent with the above *in situ* global DNA cytosine modification results (Figure 1D). In addition, there was extensive DNA methylation heterogeneity in tumor cells (Supplementary Figure S7A), as reported (Mazor et al., 2016; Lin et al., 2017; Zhang et al., 2019; Marusyk et al., 2020). Notably, there was also strong DNA methylation heterogeneity among LCSL samples (Supplementary Figure S7B). The cluster analysis of the DNA methylation pattern (Figure 3B) clearly illustrated that HCC and CCA cells were clustered together, and different samples in the paratumor were clustered together. Intriguingly, LCSL cells were clustered with paratumor cells. Thus, these results suggest that, for DNA methylation, LCSL cells were also more similar to paratumor cells than to HCC and CCA cells, consistent with the WES analysis shown above (Figures 2C,H).

By comparing the differentially methylated regions (DMRs) of distinct sample types, we found that LCSL cells displayed limited variance with paratumor cells but had greater variance with HCC and CCA cells, which is consistent with the global DNA methylation pattern (Figure 3B). Specifically, 10.1% DMRs were found between LCSL cells and PDR cells, while 27.1% or 32.3% DMRs were found between LCSL cells and HCC or CCA cells (Figure 3C and Supplementary Table S5).

Formalin-fixed, paraffin-embedded (FFPE) tissue resources are far richer than frozen tissues. We chose an FFPE HCC-CCA specimen (P4, Supplementary Table S1) and conducted WGBS analysis based on the CIL-seq method (Figure 3F). Although the quality of the sequencing data was much lower than that of frozen tissues (Supplementary Table S2), we still observed that the overall methylation level and DNA methylation pattern of LCSL cells were similar to those of PDR cells (Figures 3E,F), consistent with the results of patient P2. Taken together, these results suggest that in the global pattern of DNA methylation, very large methylation changes occurred in HCC and CCA cells compared with paratumor cells, whereas there were fewer differences in DNA methylation patterns between LCSL cells and paratumor cells.

### DNA Methylation Changes in LCSL Cells Correspond to a Chromatin Restriction State

**FIGURE 4:**
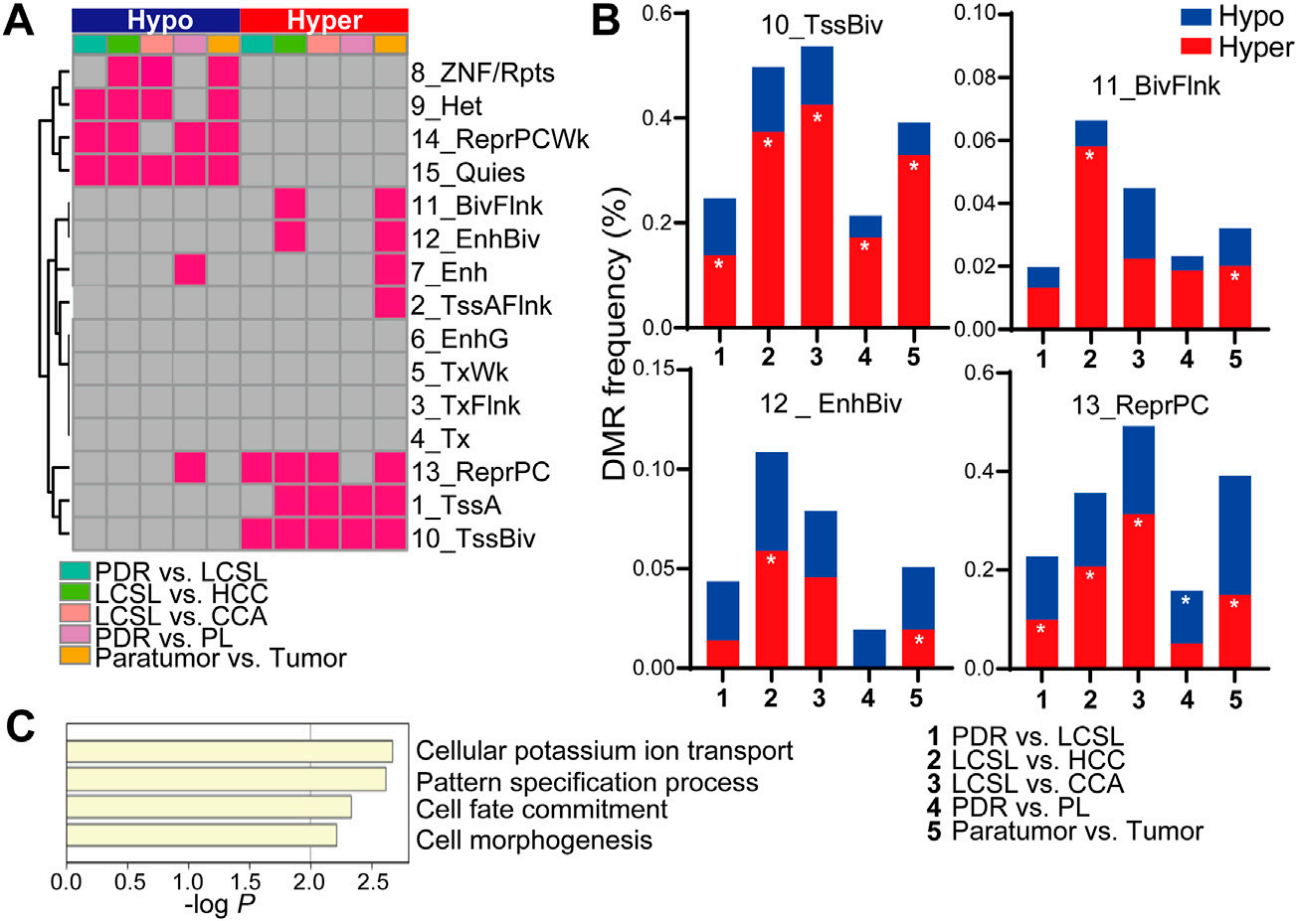
DNA methylation changes in LCSL cells correspond to a chromatin restriction state. **(A)** Significant DMR distribution in different chromatin states. We used a 15-state model consisting of eight active states and seven repressed states, as suggested in reference (Roadmap Epigenomics et al., 2015). Each red square represents significant enrichments of hypermethylated or hypomethylated DMRs in the latter cell type (Q-value < 0.01). **(B)** DMR frequency between different cell types in different chromatin states. Both hypomethylation and hypermethylation DMR frequencies between the two indicated cell types in the chromatin states 10_TssBiv, 11_BivFlnk, 12_EnhBiv and 13_ReprPC are shown. DMR frequency = DMR tiles in the chromatin state/all common identified tiles between the two cell types × 100%. The symbol * represents significant DMR enrichment of hypomethylation or hypermethylation in the latter cell type, as shown in **(A)**. **(C)** The functions of genes corresponding to the hypermethylated DMRs between PDR and LCSL cells in the chromatin states 10_TssBiv and 13_ReprPC.

We next examined the distribution region of DMRs in the genome of P2 samples. Overall, there were significantly fewer regions with prominent DNA methylation changes in LCSL cells than in HCC and CCA cells (Supplementary Table S6). According to the 15-state model of chromatin (REC et al., 2015), the significant methylation changes in HCC and CCA cells were closely related to the methylation level of the 15 chromatin states in normal cells. Chromatin states with significant demethylation occurred on those initially exhibiting a hypermethylated state in normal cells (REC et al., 2015), including the 9_Het and 15_Quies chromatin states, and chromatin states with significant hypermethylation occurred on those initially exhibiting a hypomethylated state in normal cells (REC et al., 2015), including the 1_TssA, 10_TssBiv, 11_BivFlnk and 12_EnhBiv states (Figures 4A,B and Supplementary Figure S7C). However, substantially fewer methylation changes occurred in these chromatin states in LCSL cells, which is consistent with the minor change in the global methylation level in LCSL cells (Figure 3A).

Remarkably, we found that a few crucial chromatin regions in LCSL cells had the same DNA methylation tendency as in HCC and CCA cells. Significant hypermethylation changes in repressive Polycomb group (PcG) protein-marked regions (including the chromatin states 10_TssBiv, 11_BivFlnk, 12_EnhBiv and 13_ReprPC) occurred in HCC and/or CCA cells (Figures 4A,B), which is consistent with previous findings that PcG-marked genes in both embryonic and adult progenitor systems have a higher chance of becoming hypermethylated in cancers (Easwaran et al., 2012). In addition, we also found that two of these PcG-marked regions had already undergone significant changes in LCSL cells compared with PDR cells, including in the bivalent state 10_TssBiv and the chromatin state 13_ReprPC (Figure 4B). The functions of those genes corresponding to these hypermethylated regions in LCSL cells are mainly involved in cell differentiation and fate determination (Figure 4C and Supplementary Table S7), and more than half of them (20/32) also correspond to bivalent genes in the human fetal liver (Yan et al., 2016), such as *APC2* (Supplementary Table S7), a homologue of the *APC* tumor suppressor (van Es et al., 1999). Therefore, hypermethylation changes in these regions in LCSL cells might result in a restrictive chromatin state that can block a differentiation program (Flavahan et al., 2017). Taken together, these results suggest that although the DNA methylation changes in LCSL cells were much less than those in HCC and CCA cells, the changes in LCSL cells might also result in a restrictive chromatin state, which might block or inhibit a normal differentiation program of these cells, resulting in the persistent abnormal proliferation of the cells.

### Only Minor Genetic and Epigenetic Changes in LCSL Cells Compared With Other Tumor Parenchymal Cells in HCC

**FIGURE 5:**
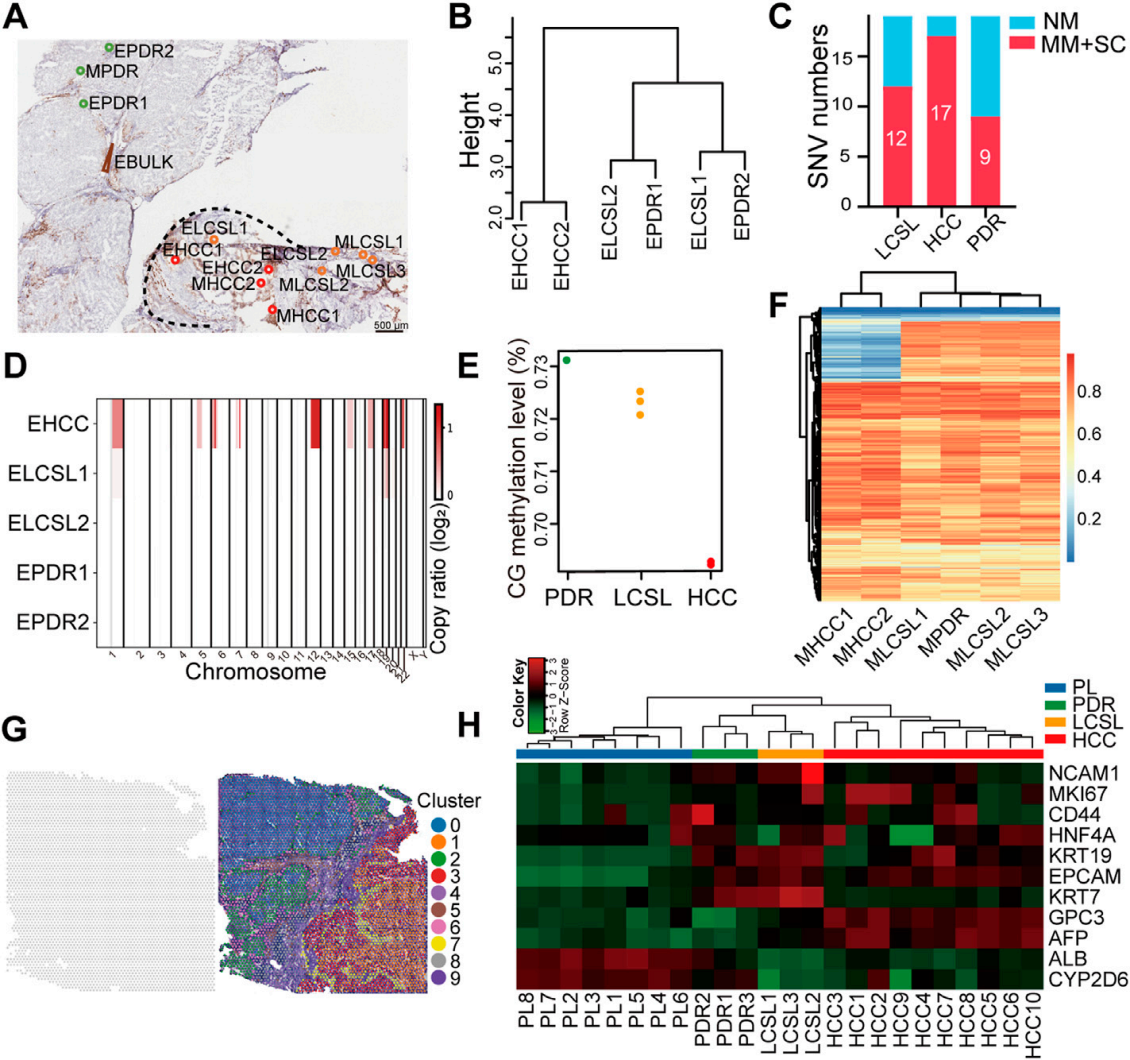
Only minor genetic and epigenetic changes in HCC LCSL cells. **(A)** Locations and names of the samples used for CIL-seq. Different types of cells were captured by LCM from a frozen section of specimen P7 after the section was immunostained with anti-EpCAM. The dotted lines in the figure indicate the boundaries between the tumor and the paratumor, which were determined by IHC staining and tissue/cell morphology under microscope. **(B)** SNV cluster analysis of the different types of cell samples. **(C)** The number of missense mutations (MM) and stop codon changes (SC) identified in different cell types. NM, no mutation. **(D)** Amplified CNVs in different cell samples. One sample (EHCC2) with low WES coverage is not shown. **(E)** The global DNA methylation levels of six different types of cells. Each dot represents one sample. **(F)** DNA methylation profiles of six cell samples. Each row is a 300-bp window that is covered by at least three bases (coverage ≥3) in all samples. **(G)** Unbiased clustering of all ST spots of P7 tissues. K-Means cluster analysis, K = 10. **(H)** Cluster analysis of the expression patterns of the selected genes in different spots, as shown in Supplementary Figure S7D.

HCC is a major type of PLC. We collected frozen tissues and prepared sections from 23 PLC cases (Supplementary Table S1). One of them (patient P7) was pathologically diagnosed with HCC and had obvious EpCAM-positive LCSL cells. We performed WES and WGBS analysis of this case with CIL-seq (Figure 5A and Supplementary Table S2). The results showed that, except for the genetic and epigenetic heterogeneity in the LCSL cells, the genetic difference (SNV and CNV) or DNA methylation pattern difference between LCSL cells and PDR cells was small (Figures 5B–F), consistent with the analysis results of patients P2 and P4. Interestingly, among the minor genetic changes in LCSL cells, we found a stop gain mutation in the driver gene *MGA* (Bailey et al., 2018), which also appeared in an HCC sample (Supplementary Table S3). *MGA* is significantly mutated in lung adenocarcinoma and participates in the negative regulation of *MYC* (Llabata et al., 2020). Thus, *MGA* mutation in certain LCSL cells may confer them a proliferative and evolutionary advantage.

Finally, we aimed to determine whether the RNA expression profiles of LCSL cells are similar to those of paratumor liver cells, since the genetic and epigenetic changes in LCSL cells are not significant. We performed a spatial transcriptomic (ST) analysis of P7 frozen tissues. We generated 4493 spot transcriptomes at a medium depth of 14,455 UMIS/spot and 4064 genes/spot (Supplementary Figure S8A), which can clearly distinguish tumor and paratumor cells (Supplementary Figure S8B). In general, there were large RNA expression differences between the tumor and paratumor cells (Supplementary Figure S8C). Further cluster analysis showed that the tumor cells had extensive heterogeneity (Figure 5G). Because of the small proportion of LCSL cells, we manually selected all LCSL cell spots in the tumor tissues and PDR cell spots in the paratumor tissues (Supplementary Figures S8D,E). In addition, we randomly selected some spots of tumor or paratumor parenchymal cells. Cluster analysis of the whole expression profiles showed that LCSL cells and PDR cells were clustered together (Supplementary Figure S8F). The cluster analysis of the expression profiles of 11 liver-related genes of these spots showed similar results (Figure 5H), indicating that there was only a small difference in RNA expression patterns between LCSL cells and PDR cells, consistent with the results of the genetic and epigenetic changes.

## Discussion

**FIGURE 6:**
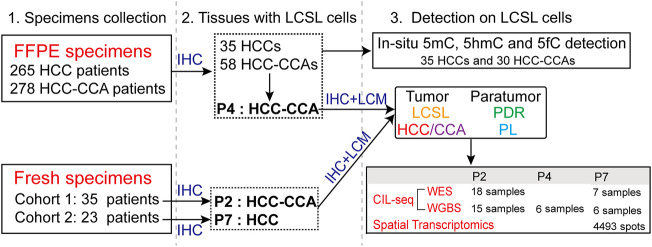
An outline of samples used and experiments performed in this study. FFPE and fresh specimens of primary liver cancer patients were collected and screened for LCSL cells by EpCAM IHC staining and tissue/cell morphology. Spatial mutiomics analysis, including *in-situ* detection of DNA cytosine modifications (5mC, 5hmC and 5fC), WES and WGBS analysis (for P2, P4 and P7 tissues) by CIL-seq, and spatial transcriptomics (for P7 tissues), was performed on those specimens with obvious LCSL cells.

In the present study, using spatial multiomics analysis (Figure 6), we provide evidence that only a few genetic and epigenetic changes occur in human LCSL cells compared with other tumor parenchymal cells. Our data showed that HCC and CCA cells have undergone considerable genetic and epigenetic changes that we usually see in human cancers, but only minor genetic and epigenetic changes have occurred in LCSL cells, although some of the changes may be important for CSC formation.

In the past few years, there have been many studies on the genetics and DNA methylation of various human cancers, including various types of liver cancer, and found a wide range of changes in tumor tissues. However, these studies were mainly focused on local bulk tumor tissues, and there were few studies and little information about CSCs directly from patients. Because of the possible great genetic and epigenetic differences between CSCs and other tumor parenchymal cells, the detection of genetic and epigenetic changes in whole tumor tissue could not reflect what has happened in CSCs. The sequencing signals from bulk tumor samples would be dominated by major tumor parenchymal cells, rendering rare CSCs undetectable. Therefore, treatments that target most of the significant genetic and epigenetic changes (and hence the changes in RNA level and protein level) in these tumors may not be effective for CSCs because CSCs may not possess those changes.

The LCSL cells in the patients in this study harbored CSC characteristics, although they closely resembled PDR cells in cell morphology, the expression of some key markers of liver stem cells (Supplementary Figure S1B), and the global levels of DNA cytosine modifications (Figure 1D). The CSC characteristics of LCSL cells include *1*) the expression of some commonly recognized liver stem cell markers, *2*) a higher proliferative capacity than PDR cells (Figure 1B) and *3*) histopathological (Figure 1A) and genetic evidence (Figure 2H) suggesting that LCSL cells may be the common origin of HCC and CCA cells. In addition, a very small number of key genetic or epigenetic changes, for example, *MYC* amplification (Supplementary Figure S6), were identified in these LCSL cells, but not in PDR cells. Taken together, these characteristics of LCSL cells clearly indicate that LCSL cells have characteristics of both tumor cells and stem cells and are different from paratumor PDR cells.

The proportion of CSCs or LCSL cells in some mixed HCC-CCAs is usually much higher than that in other types of tumors; therefore, such mixed HCC-CCAs can provide us with an ideal tumor object to help investigate human CSCs. At present, mixed HCC-CCA can only be diagnosed by pathological examination after surgery, and the proportion of mixed HCC-CCA in PLC is very low, so it is not easy to collect many samples of mixed HCC-CCA for frozen sections. However, DNA methylation data obtained by the CIL-seq method from FFPE samples (Figures 3D–F) could also provide useful information to support our conclusion. In addition, *in situ* detection of the global level of cytosine modifications (Figure 1D) and the results obtained from HCC tissues (Figure 5) all support our conclusion that only minor genetic and epigenetic changes occurred in LCSL cells.

The epigenetic changes in LCSL cells of P2 patient are interesting, although the changes are minor compared with those in HCC cells or CCA cells. Since there are only minor genetic changes in LCSL cells, we propose that epigenetic changes in LCSL cells may be important for cancer initiation and evolution. Through restricting changes in gene activity, chromatin structures and regulators may increase the heights of energy walls between cell states and resist changes in cell identity (Yuan et al., 2019). Our results showed that a few crucial chromatin regions in LCSL cells, such as repressive PcG protein-marked regions, had a tendency of hypermethylation. Hypermethylation changes in these regions might result in a restrictive chromatin state in LCSL cells, which can block a differentiation program (Yuan et al., 2019). Thus, epigenetic changes in LCSL cells might block or inhibit the normal differentiation program of these cells, resulting in persistent abnormal proliferation and evolution of the cells.

In summary, our findings revealed the important genetic and epigenetic properties and changes in the LCSL cells of liver cancer patients. In particular, we found only minor genetic and epigenetic changes in LCSL cells compared with other tumor parenchymal cells. Further studies are needed to determine whether such properties and changes are common in CSCs in patients with other types of cancer. Identifying few but key genetic and epigenetic changes in both cancer stem-like cells (or CSCs) and other tumor parenchymal cells of each specific cancer patient, such as *MYC* amplification in patient P2 or *MGA* mutation in patient P7 in this study, could help design more effective methods to treat human cancer in the future.

## Data Availability

The datasets presented in this study can be found in online repositories. The raw sequencing data were deposited in the National Omics Data Encyclopedia under accession number OEP000991 (https://www.biosino.org/node/project/detail/OEP000991).

## Abbreviations

AFP, alpha fetal protein; ALB, albumin; CK19, cytokeratin 19; CNA, copy number alteration; CSC, Cancer stem cell; DMR, differentially methylated region; EpCAM, epithelial cell adhesion molecule; 5fC, 5-formylcytosine; FFPE, formalin-fixed, paraffin-embedded; GO, gene oncology; HCC-CCA, hepatocellular carcinoma-cholangiocarcinoma; H&E, hematoxylin and eosin; 5hmC, 5-hydroxymethylcytosine; IHC, immunohistochemistry; LCM, laser capture microdissection; LCSL, liver cancer stem-like; 5mC, 5-methylcytosine; OCT, optimal cutting temperature; PcG, polycomb group; PDR, paratumor ductular reaction; PL, paratumor liver; PLC, primary liver cancer; SALL4, Sal-like protein 4; SNV, single nucleotide variant; ST, spatial transcriptome; UMI, unique molecular identifier; WES, whole exome sequencing; WGBS, whole genome bisulfite sequencing; WGS, whole-genome sequencing.
