# Supplementary material for: Spatial Multiomics Analysis Reveals Only Minor Genetic and Epigenetic Changes in Human Liver Cancer Stem-Like Cells Compared With Other Tumor Parenchymal Cells
Source: Front Cell Dev Biol. 2022 Feb 9;10:810687. doi: 10.3389/fcell.2022.810687 (PMC8863946; doi:10.3389/fcell.2022.810687)
Supplement: Supplementary file 1 [file DataSheet1.pdf]

Supplementary Material

Supplementary materials contain 8 Supplementary Figures and 7 Supplementary Tables. The supplementary tables are provided as excel files, separately.

This PDF file includes:

Figures S1-S8

Other Supplementary Materials for this manuscript include the following:

Supplementary Tables S1-S7 (Excel)

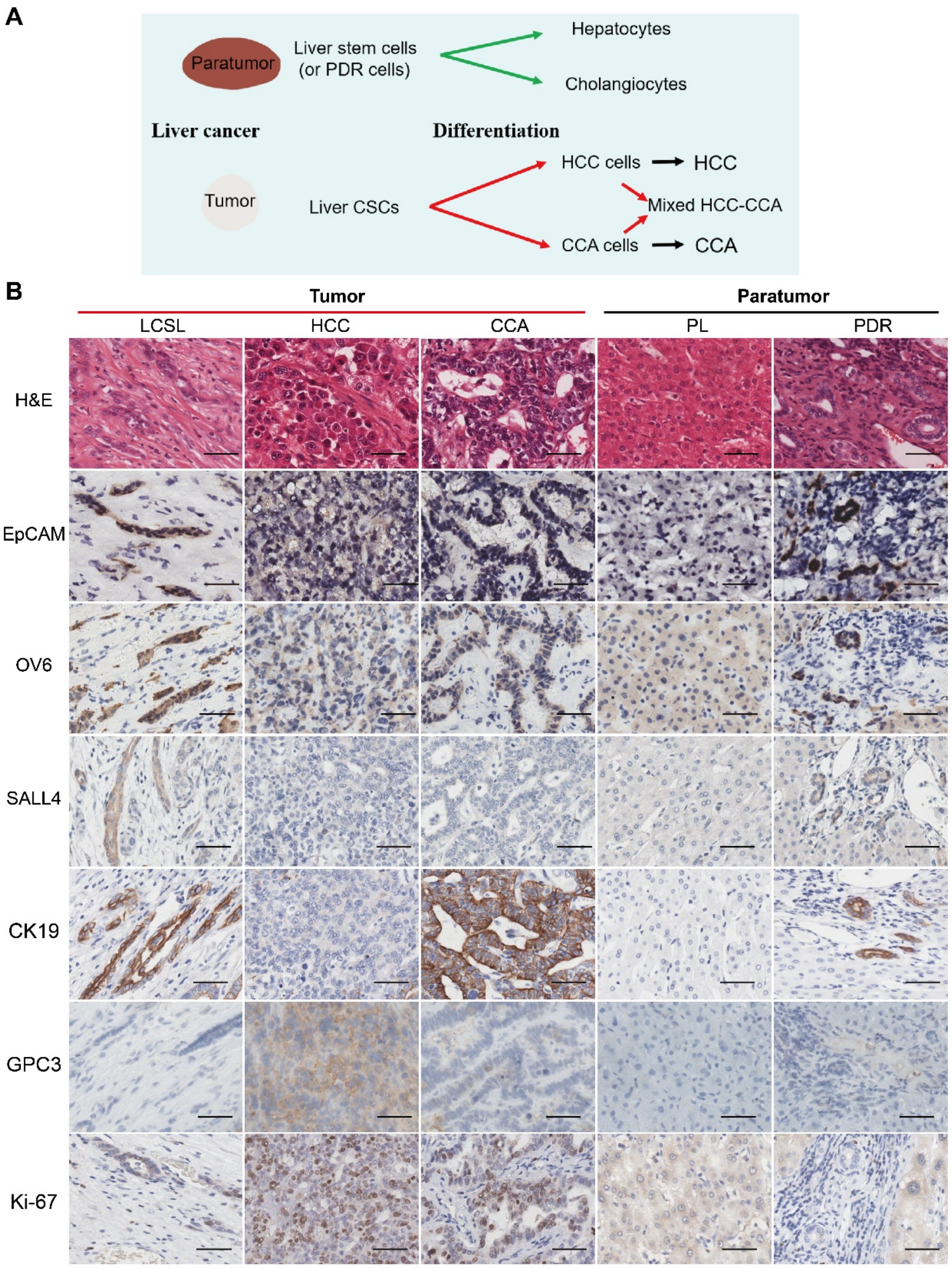

Figure S1. Liver stem cells and liver cancer stem cells (CSCs) or liver cancer stem-like (LCSL) cells in liver cancer.

(A) The diagram shows that paratumor liver stem cells or ductular reaction (PDR) cells can differentiate into hepatocytes and cholangiocytes, while liver CSCs can differentiate and evolve into hepatocellular

carcinoma (HCC) and cholangiocarcinoma (CCA) cells, which are components of HCC, CCA or mixed HCC-CCA.

(B) LCSL cells and other types of cell samples in P2 specimen. Representative hematoxylin and eosin (H&E) staining and IHC (anti-EpCAM, OV6, SALL4, CK19, GPC3 and Ki-67) staining of liver cancer resection samples, including two types of liver parenchymal cells in the paratumor: PL cells, PDR cells; and three types of liver parenchymal cells in tumor: LCSL cells, HCC cells and CCA cells. Note that HCC cells and CCA cells were also stained positively for some liver stem cell markers, including EpCAM. Thus, tissue / cell morphology is also essential and helpful for the identification of these cells. Scale bar, 50 μm.

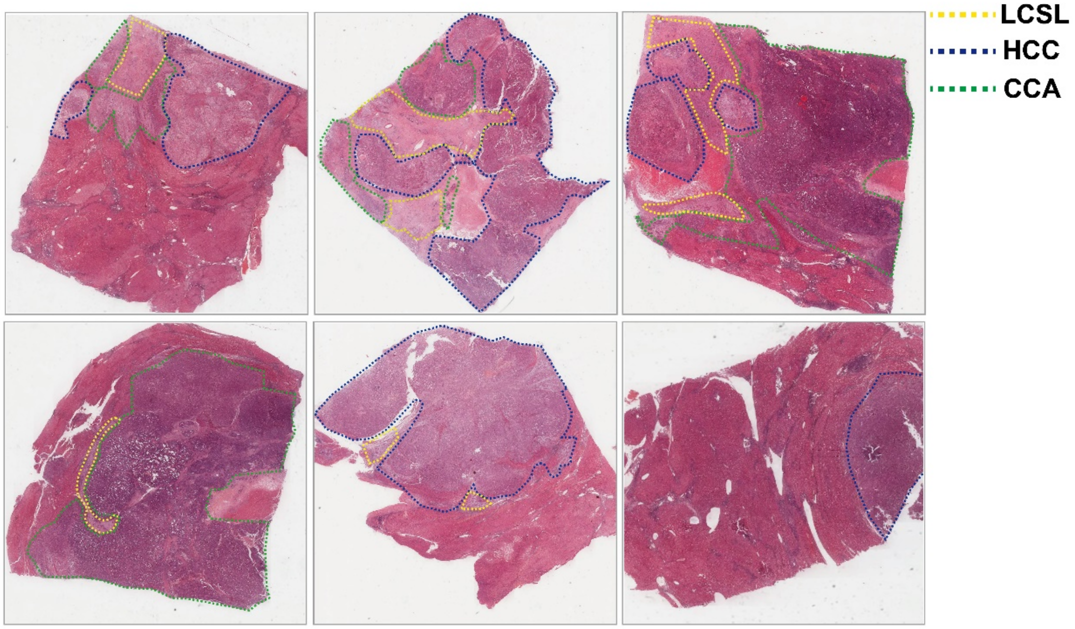

Figure S2. Three types of tumor parenchymal cells in P2 specimen.
Representative H&E staining of several different parts of the tumor. The dotted lines in the figure indicate the boundaries of different tumor parenchymal cells, which were determined by H&E staining and tissue / cell morphology under microscope. LCSL, liver cancer stem-like cells; HCC, hepatocellular carcinoma cells; CCA, cholangiocarcinoma cells.

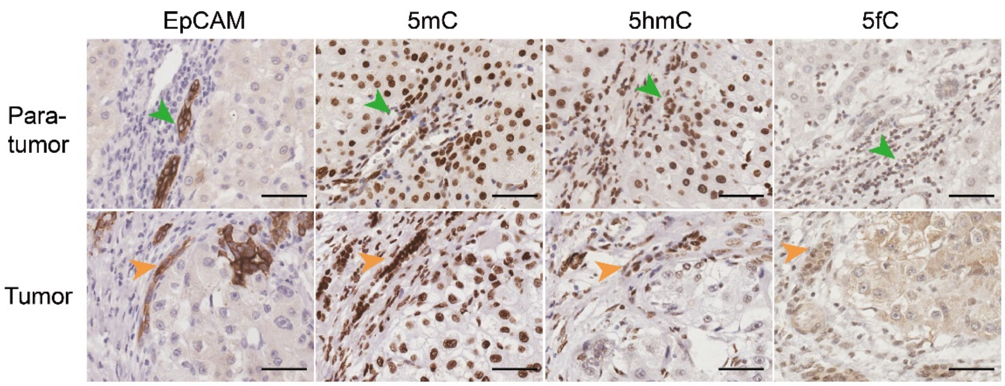

Figure S3. IHC staining of DNA cytosine modifications in liver cancer tissues.

Representative IHC staining (anti-EpCAM, 5mC, 5hmC and 5fC) of the P2 FFPE section samples. The green and yellow arrowheads indicate PDR and LCSL cells, respectively. Scale bar, 50 μm.

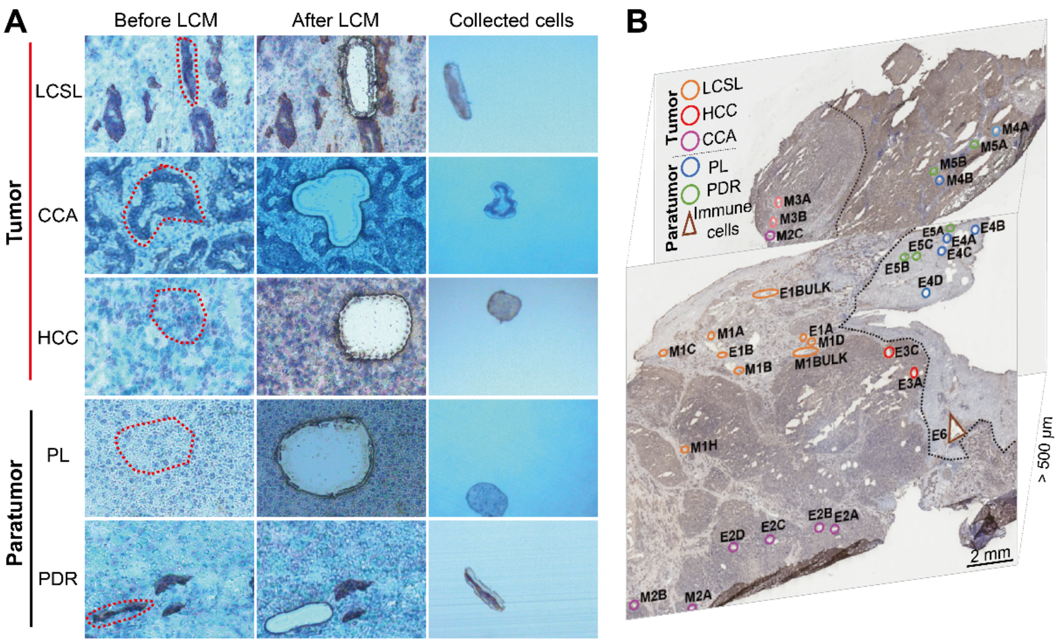

C

Location	Type	WES samples	WGBS samples
Tumor	LCSL	E1A; E1B; E1BULK	M1A; M1B; M1C; M1D; M1H; M1BULK
	CCA	E2A; E2B; E2C; E2D	M2A; M2B; M2C
	HCC	E3A; E3C	M3A; M3B
Paratumor	PL	E4A; E4B; E4C; E4D	M4A; M4B
	PDR	E5A; E5B; E5C	M5A; M5B
	Immune cells	E6	

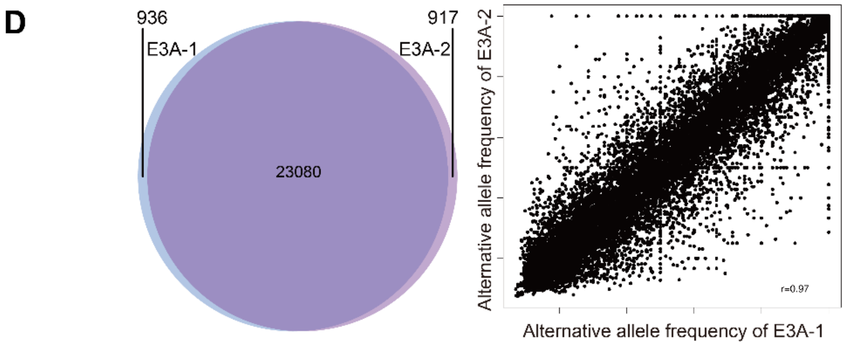

Figure S4. Cell samples from P2 for sequencing.

(A) Examples of fine LCM cutting.

(B) Locations and names of the samples of patient P2 used for WES and WGBS analysis. The samples were collected from two frozen sections that were stained with anti-EpCAM and had a spatial distance greater than 500 μm from each other. The dotted lines indicate the boundaries between the tumor and the paratumor, which were determined by IHC staining and tissue / cell morphology under a microscope.

(C) Summary of the names of the samples in different cell types, related to Fig. 1D. Each liver cell sample had one clone or cluster with 20 to 40 cells except for two LCSL cell samples (E1BULK and M1BULK)

which was a multiple mixture of approximately 10 LCSL cell clusters, and there was a certain spatial distance (> 200 μm) among the samples.

(D) Repeatability of WES for the sample E3A (a sample of HCC cells). Left, comparison of germline variants from two technical replicates (E3A-1 and E3A-2); right, correlation plot of the alternative allele frequency between the two replicates.

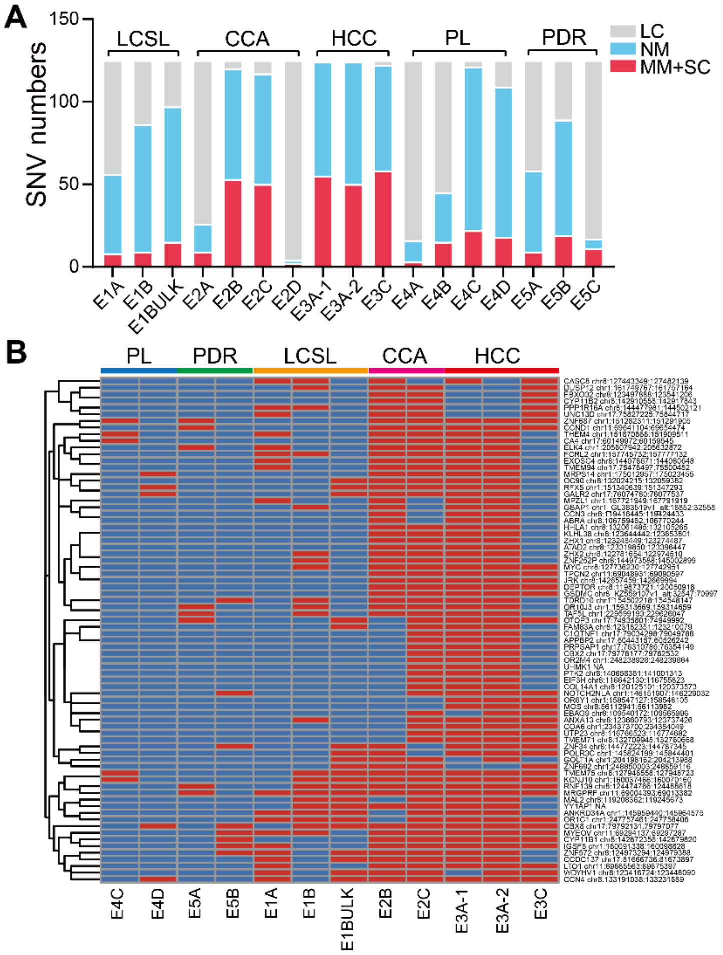

Figure S5. WES analysis of different cell types of P2.

(A) The number of SNVs identified in different cell types of P2. LC, low coverage; NM, no mutation; MM, missense mutations; SC, stop codon changes.

(B) Amplified CNA genes in different samples. The red color represents amplification. Samples with low WES coverage are not shown.

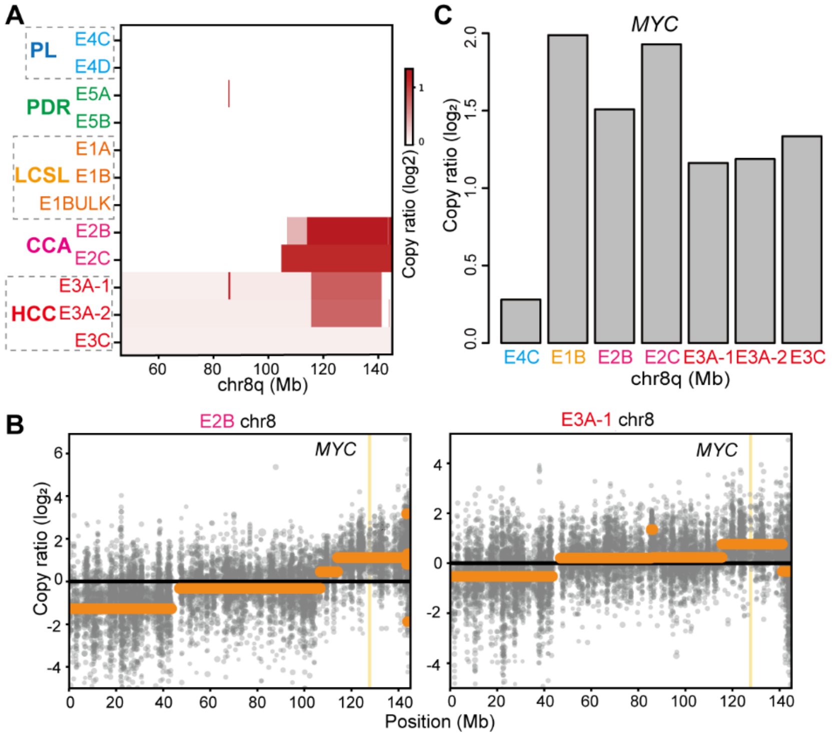

Figure S6. The amplification pattern of the 8q region, which includes the *MYC* gene, in different samples.

(A) Heatmap illustrating the copy number ratio of chromosome 8q ($log_2R > 0$).

(B) Example of copy number amplification in CCA (E2B) and HCC (E3A-1) cells. Points and lines represent bin-level and segment-level copy number ratio.

(C) Copy number ratio of *MYC* gene. Only samples with $log_2R > 0$ are shown.

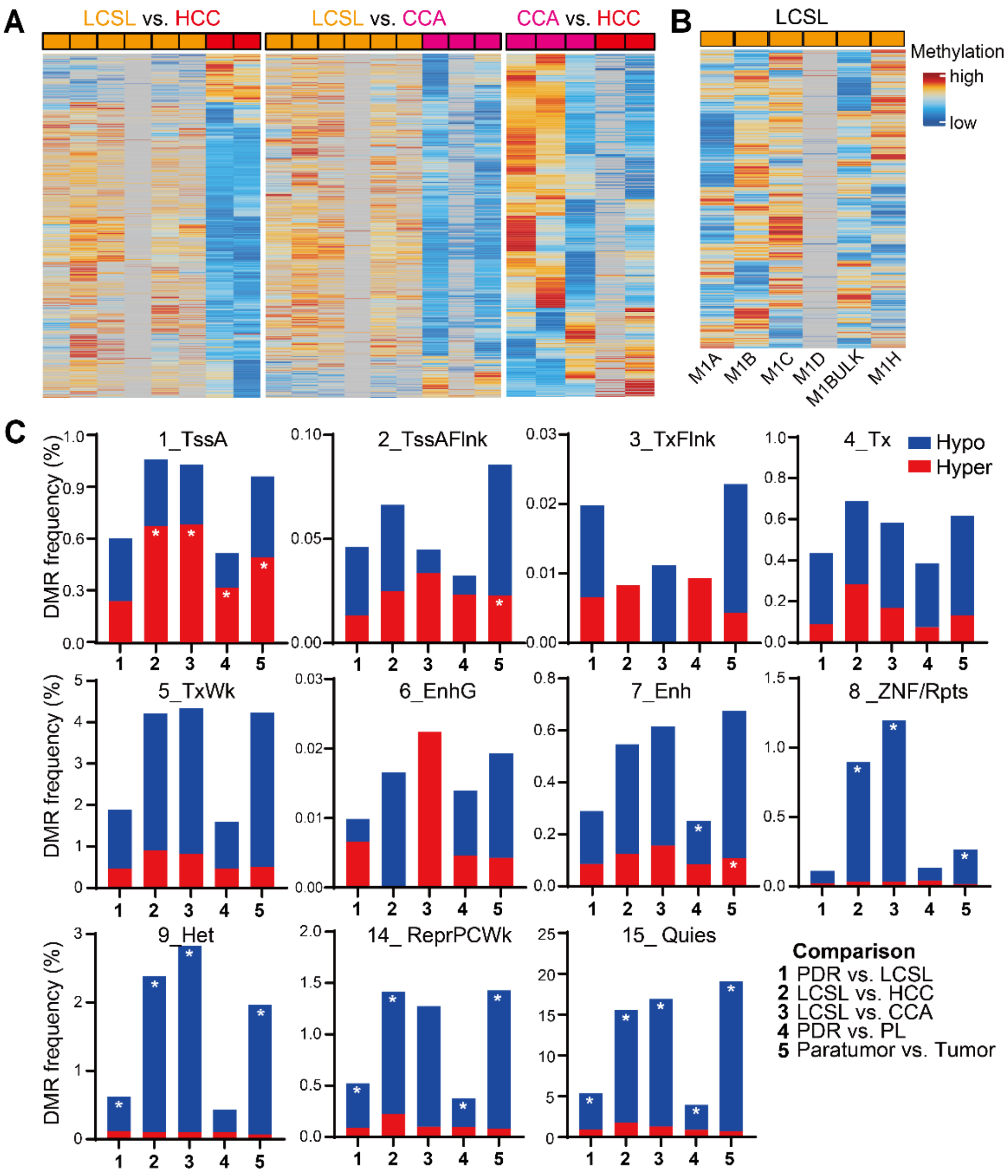

Figure S7. DNA methylation changes in different cell types of P7.

(A) DMRs between two tumor cell types. The DNA methylation status in DMRs in each sample are shown. (B) DNA methylation profiles of 6 LCSF cell samples. Each row is a 300bp window that is covered by at least 3 bases in more than 3 samples. The grey color in (A) and (B) represents uncovered regions.

(C) DMR frequency between different cell types in different chromatin states. Similar to Fig. 4B, both hypermethylation and hypomethylation DMR frequency between any two indicated cell types in all 15 chromatin states except for states 10_TssBiv, 11_BivFlnk, 12_EnhBiv and 13_ReprPC are shown. DMR frequency = DMR tiles in the chromatin state / all common identified tiles between the two cell types × 100%. The symbol * represents significant DMR enrichment between the two cell types as in Fig. 4A.

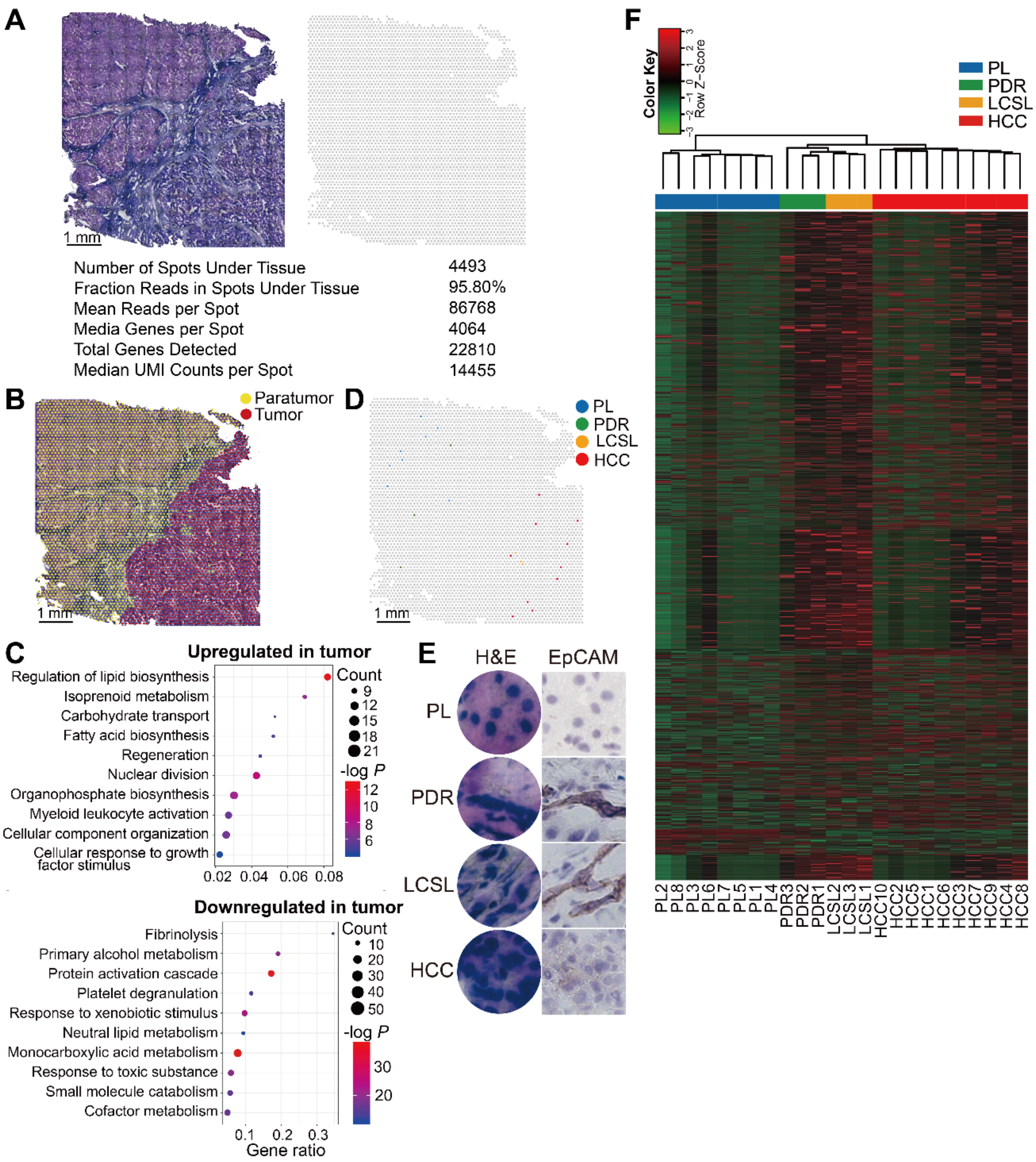

Figure S8. Similar RNA expression profiles between LCSL and PDR cells revealed by spatial transcriptomics.

(A) H&E staining of a frozen tissue section of P7 for ST and quality summary of ST.

(B) Unbiased clustering of ST spots. K-Means cluster analysis, K = 2.

(C) Dot plot of GO terms for top 200 significantly up- or down-regulated genes between the tumor and the paratumor.

(D) LCSL cell spots as well as other cell spots selected for analysis.

(E) Representative H&E (directly from the ST spots) and IHC (anti-EpCAM) staining of PL, PDR, LCSL and HCC cell spots.

(F) Cluster analysis of the global RNA expression profiles of the spots in (D).
